# Supplementary figures and images for: Use of Transabdominal Ultrasound for the detection of intra-peritoneal tumor engraftment and growth in mouse xenografts of epithelial ovarian cancer
Source: PLoS One. 2020 Apr 29;15(4):e0228511. doi: 10.1371/journal.pone.0228511 (PMC7190129; doi:10.1371/journal.pone.0228511)

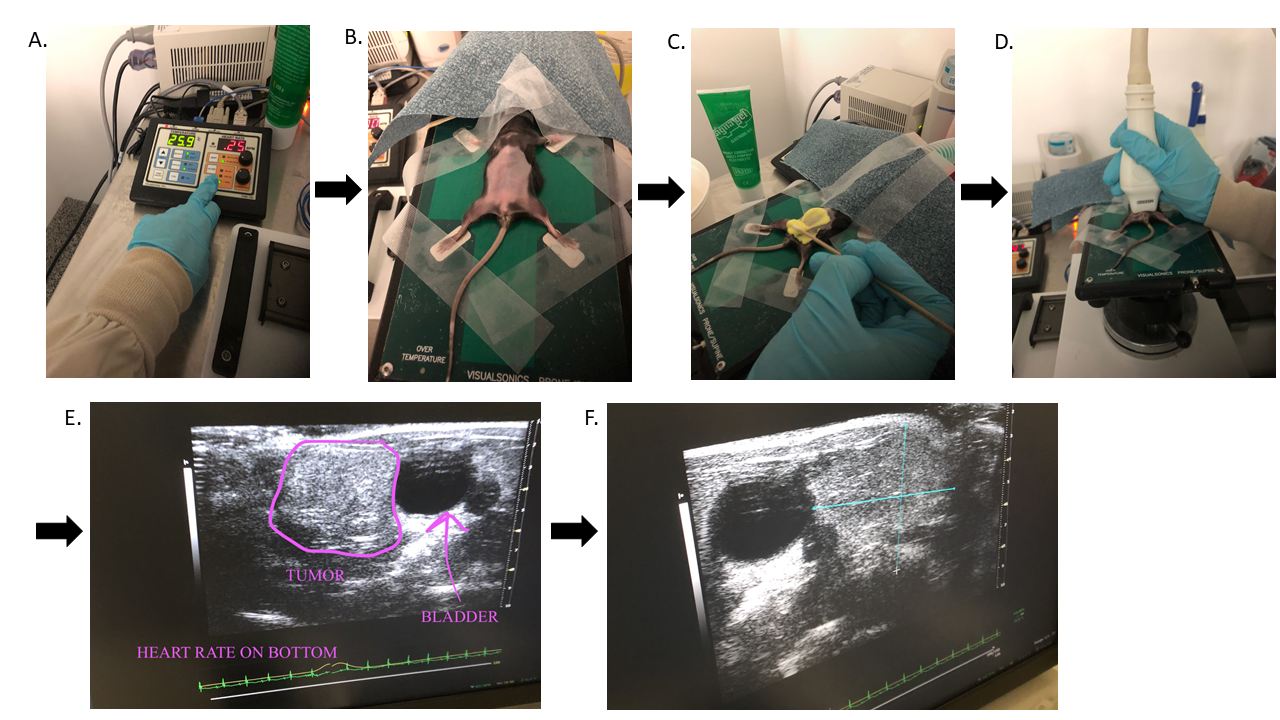

Supplement: S1 Fig — (TIF) [file pone.0228511.s001.tif]

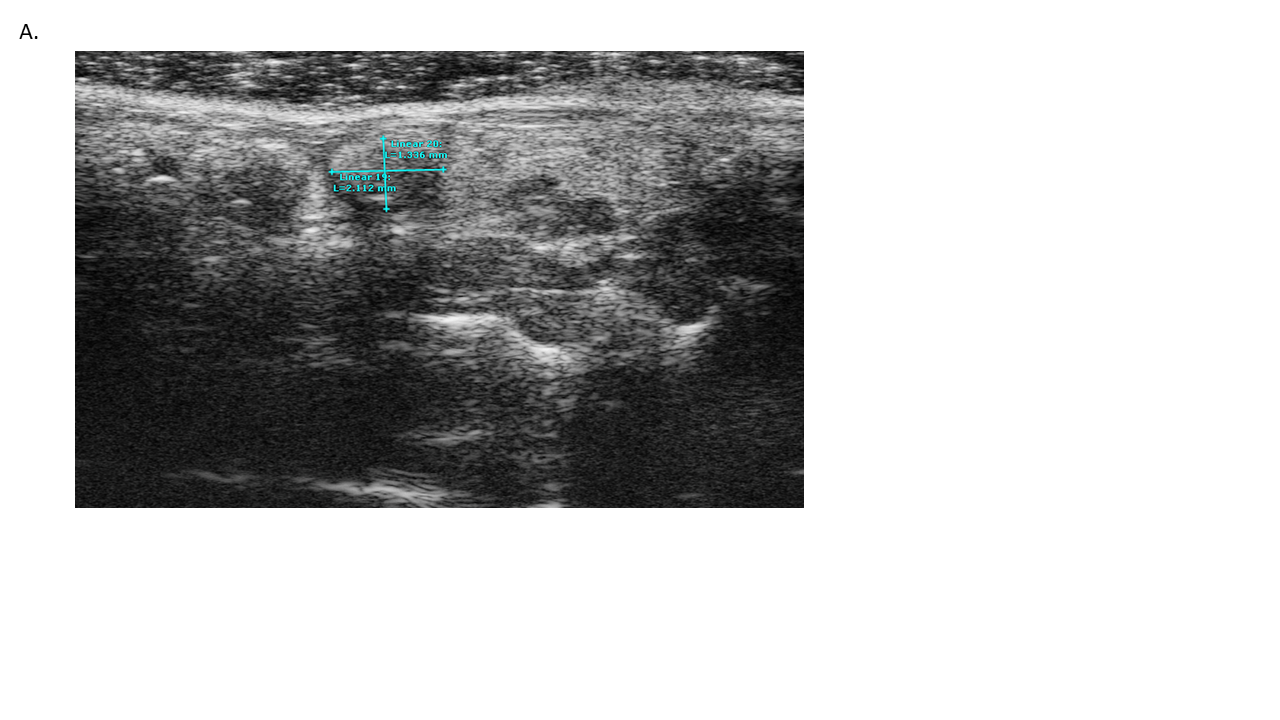

Supplement: S2 Fig — (TIF) [file pone.0228511.s002.tif]
